# Long-range superharmonic Josephson current and spin-triplet pairing correlations in a junction with ferromagnetic bilayers

**DOI:** 10.1038/srep21308

**Published:** 2016-02-19

**Authors:** Hao Meng, Jiansheng Wu, Xiuqiang Wu, Mengyuan Ren, Yajie Ren

**Affiliations:** 1 Department of Physics, South University of Science and Technology of China, Shenzhen, 518055, China; 2 School of Physics and Telecommunication Engineering, Shaanxi University of Technology, Hanzhong 723001, China; 3 National Laboratory of Solid State Microstructures and Department of Physics, Nanjing University, Nanjing 210093, China; 4 School of material science and technology, Harbin university of science and technology, Harbin 150080, china

## Abstract

The long-range spin-triplet supercurrent transport is an interesting phenomenon in the superconductor/ferromagnet (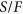) heterostructure containing noncollinear magnetic domains. Here we study the long-range superharmonic Josephson current in asymmetric 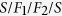 junctions. It is demonstrated that this current is induced by spin-triplet pairs 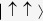 − 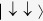 or 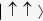 + 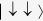 in the thick 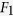 layer. The magnetic rotation of the particularly thin 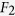 layer will not only modulate the amplitude of the superharmonic current but also realise the conversion between 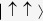 − 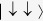 and 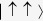 + 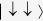. Moreover, the critical current shows an oscillatory dependence on thickness and exchange field in the 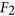 layer. These effect can be used for engineering cryoelectronic devices manipulating the superharmonic current. In contrast, the critical current declines monotonically with increasing exchange field of the 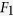 layer, and if the 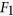 layer is converted into half-metal, the long-range supercurrent is prohibited but 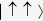 still exists within the entire 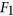 region. This phenomenon contradicts the conventional wisdom and indicates the occurrence of spin and charge separation in present junction, which could lead to useful spintronics devices.

Superconductor/ferromagnet (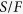) hybrid structure has recently attracted considerable attention because of the potential applications in spintronics and quantum information^1^,^2^,^3^ as well as the display of a variety of unusual physical phenomena^4^,^5^,^6^,^7^. In general, if a weak *F* is adjacent to an s-wave *S* and there is no interfacial spin-flip scattering, the normal Andreev reflection will generate at 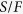 interfaces. The process involves an electron incident on the 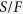 interface from the *F* at energies less than the superconducting energy gap. The incident electron forms a Cooper pair in the *S* with the retroreflection of a hole of opposite spin to the incident electron. Consequently, the conventional spin-singlet Cooper pair decays at a short range in ferromagnetic region. In 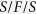 Josephson junctions with homogeneous magnetization, through the normal Andreev reflection occurring at two 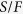 interfaces, a Cooper pair is transferred from one *S* to another, creating a supercurrent flow across the junction^8^. As a consequence of the exchange splitting of the Fermi level of the *F*, the Cooper pair shows an oscillatory manner superimposed on an exponential decay in the *F*. Correspondingly, the Josephson current displays a damped oscillation with increasing the thickness or the exchange field of the *F*, leading to the appearance of the so-called “0-*π* transition”^1^,^2^. In general, the normal Andreev reflection will be suppressed by the exchange field of the *F*, so the Josephson current just can transport a short distance.

In contrast, if one insert a thin spin-active *F* layer with noncollinear magnetization into the 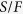 interface, it is found that the noncollinear magnetization can lead to a spin-flip scattering, then the reflected hole has the same spin as the incident electron, which is identified as anomalous Andreev reflection. When this reflection takes place at two 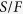 interfaces, the parallel spin-triplet Cooper pairs 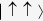 are generated in the central *F* layer and can penetrate into *F* layer over a long distance unsuppressed by the exchange interaction, so that the proximity effect is enhanced. The induced long-range current manifests itself as a large first harmmonic (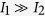) in the spectral decomposition of the Josephson current-phase relation 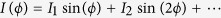 8.

It is worth to point that, if the central *F* layer is converted into fully spin-polarized half-metal, in which electronic bands exhibit insulating behavior for one spin direction and metallic behavior for the other, the normal Andreev reflection will be inhibited completely due to inability to form a pair in the *S* and impossibility of single-particle transmission. However, the strength of the anomalous Andreev reflection can not be strongly influenced by the spin-polarization of the *F*, and the transport processes of 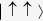 (or 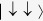) in the *F* region will continue to take place. In response, several different inhomogeneous configurations have been proposed for studying such enhanced proximity effect^9^,^10^,^11^,^12^,^13^,^14^,^15^. The corresponding experiments have proved these physical process and observed the strong enhancement of the long-range spin-triplet supercurrents^16^,^17^,^18^,^19^,^20^,^21^.

Different from the configurations mentioned above, it has proposed a long-range proximity effect develops in highly asymmetric 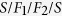 junction composed of thick 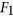 layer and particularly thin 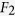 layer with noncollinear magnetizations at low temperatures^22^,^23^,^24^. This effect arises from two normal Andreev reflections occurred at normal 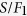 interface and two anomalous Andreev reflections at spin-active 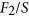 interface. The long-range spin-triplet correlations in this junction give the dominant second harmonic (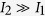) in current-phase relation 23, which is known as superharmonic Josephson current^22^. Recently, Iovan *et al.*^25^ experimentally observed the long-range supercurrent through above junction. This second harmonic can be manifested as half-integer Shapiro steps that can be experimentally observed^26^, and the two times smaller flux quantum will be obtained, leading to more sensitive quantum interferometers (SQUIDs)^27^. It should be stressed that refs 22, 23, 24 did not discuss the difference of long-range triplet pairing fashion between asymmetric 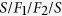 junction and symmetric 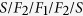. Moreover, it is high desirable to clarify the effect of the misorientation angle on the triplet pairing correlations in the 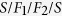 junction, as well as the influence of the thickness and the exchange field in two ferromagnetic layers on the Josephson current and the long-range spin-triplet correlations.

In this work, we study the relation between the long-range superharmonic Josephson current and the spin-triplet pairing correlations in 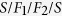 junction. It is proposed that the superharmonic Josephson current is induced by the spin-triplet pairs 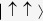 − 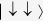 or 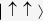 + 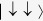 in the long 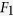 layer. The variation of the misorientation angle between two magnetizations will not only turn the amplitude of the superharmonic current but also realize the conversion between 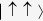 − 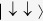 and 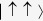 + 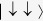. This can be used to control the superharmic current and the pairing fashion in the 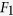 layer through modulating the magnetic structure of the 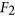 layer. Besides, the critical current shows an oscillatory dependence on the thickness and exchange field of the highly thin 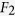 layer. These effect can be used for engineering cryoelectronic devices manipulating spin-polarized supercurrent. In contrast, the critical current decreases monotonically with increasing exchange field of the 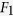 layer. Specifically, if the 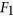 layer is converted into half-metal, the long-range Josephson current will be completely prohibited, but 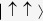 still exist in 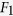 region. This phenomenon indicates the occurrence of spin and charge separation in present 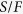 junction which could lead to useful spintronics devices. These results also contradict the traditional view: the long-range Josephson current is determined by the parallel spin-triplet pairs in the multilayer junction with noncollinear magnetization alignment between ferromagnetic layers. At last, it is also found that the magnetization of the 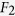 layer will bring about a same direction magnetization in the 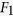 layer on condition that the magnetic moment of the 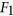 layer is weak.

To be more precise, we consider the Josephson junction consists of two s-wave superconducting electrodes and ferromagnetic bilayer with noncollinear magnetizations. The schematic picture of the 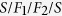 device is presented in Fig. 1. One assume that the transport direction is along the *y* axis, and the system satisfies translational invariance in the *x-z* plane. The thicknesses of 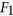 layer and 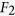 layer are 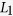 and 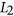, respectively. The exchange field 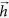 due to the ferromagnetic magnetizations in the 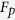 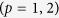 layer is described by 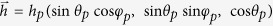. Here 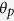 is the tilt angle from the *z* axis, and 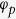 is the horizontal angle respect to *x* axis.

## Results

Based on the extended the Blonder-Tinkham-Klapwijk (BTK) approach^28^,^29^,^30^,^31^, the dc Josephson current in the 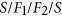 junction can be expressed as follows


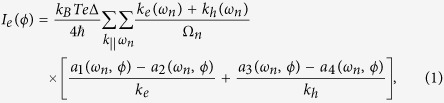


where 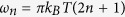 are the Matsubara frequencies with 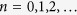 and 
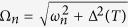
. 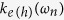 are the perpendicular components of the wave vectors for electron-like (hole-like) quasiparticles in superconducting regions, and 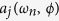 with 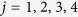 are the scattering coefficients of the normal Andreev reflection under the condition of four different incoming quasiparticles, electron-like quasiparticles (ELQs) and hole-like quasiparticles (HLQs) with spin up and spin down. Then the critical current is derived from 
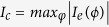
.

By applying the Bogoliubov’s self-consistent field method^32^,^33^, the triplet pair amplitudes are defined as follows^34^:


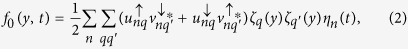



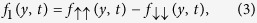



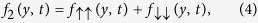


where 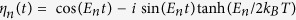, and equal-spin pair amplitude will be denoted by 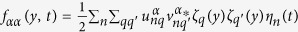. The singlet pair amplitude writes as 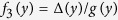. In this paper, the singlet and triplet pair amplitudes are all normalized to the value of the singlet pairing amplitude in a bulk superconducting material. The LDOS is given by^34^


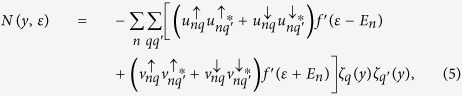


where 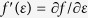 is the derivative of the Fermi function. The LDOS is normalized to unity in the normal state of the *S* material. In addition, the local magnetic moment in the 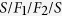 geometry has three components^34^.


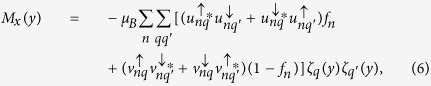



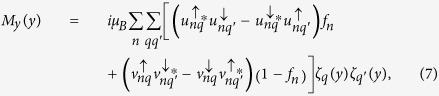



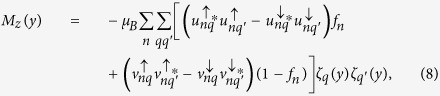


where 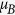 and 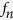 are the Bohr magneton and the Fermi function, respectively. It is convenient to normalize these components to 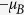.

Unless otherwise stated, in BTK approach we use the superconducting gap 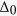 as the unit of energy. The Fermi energy is 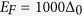, the interface transparency is 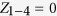 and 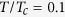. We measure all lengths and the exchange field strengths in units of the inverse of the Fermi wave vector 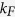 and the Fermi energy 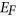, respectively. The magnetization in the 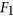 layer is fixed along the *z* direction (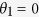, 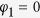), while the 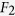 is a free layer in which the magnetization points any direction. In Bogoliubov’s self-consistent field method, we consider the low-temperature limit and take 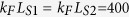, 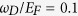. The other parameters are the same as the ones mentioned before.

## Discussion

### Superharmonic currents versus misalignment angle

**Figure 2:**
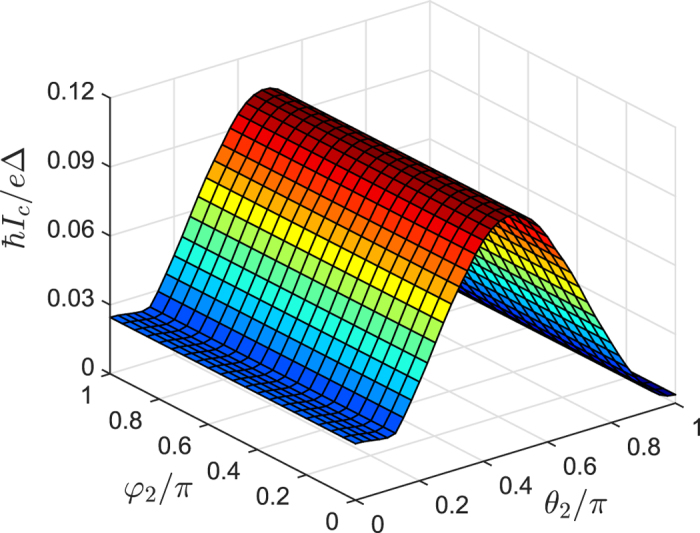
Critical current as a function of the orientation angle (*θ*_2_, *φ*_2_) of the *F*_2_ layer. Here we set 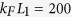, 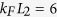, 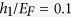, and 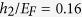.

**Figure 3:**
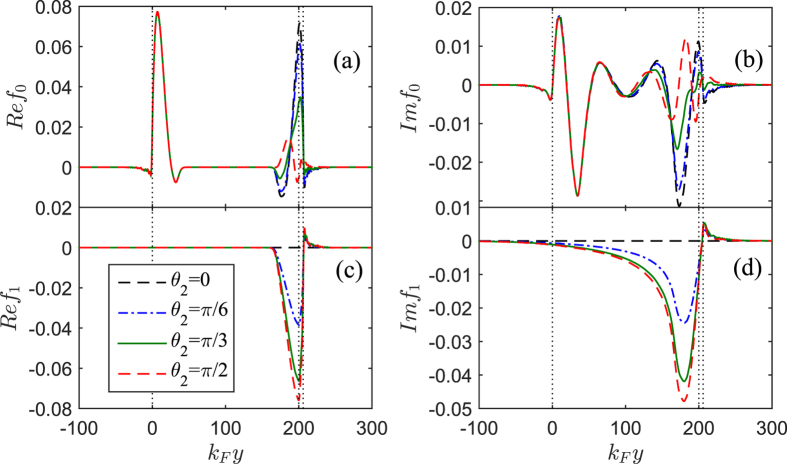
The spin-triplet pair amplitudes *f*_0_ and *f*_1_ plotted as a function of the coordinate *k*_*F*_*y* for several values of *θ*_2_ in the case of *φ*_2_ = 0. The left panels show the real parts while the right ones show the imaginary parts. The dotted vertical lines represent the location of the 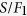, 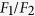 and 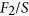 interfaces. Here 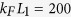, 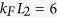, 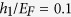, 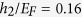, 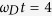, and 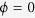. All panels utilize the same legend.

From Fig. 2 one can clearly see that the critical current reaches maximum for perpendicular magnetizations (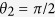) and decreases to minimum as the magnetizations are parallel (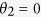) or antiparallel (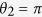) to each other. However, the variation of the angle 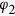 can not lead to the change of critical current while keeping 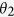 constant. It is known that characteristic variations of the critical current 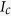 with the misaligned angles (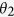, 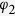) are related to the nature of pairing correlations. Figure 3 shows the spatial distribution of the spin-triplet pair amplitudes for different misalignment angle 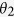 at fixed 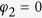. It is found that the real part of 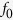 and 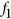 can not penetrate entire 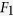 layer, but their image parts can be distributed throughout this region. With increasing 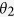, the left parts of 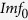 are almost unchanged, however, their right parts gradually decrease. Correspondingly, the amplitudes of 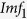 increase and turn to maximum at 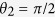. The main reason is because the *x*-projection of misaligned magnetic moment in the 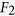 layer can generate two separate effects: spin-mixing and spin-flip scattering process^9^. The former will result a mixture of singlet pairs and triplet pairs with zero spin projection 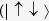 − 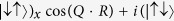 + 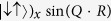, where 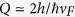, 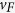 is the Fermi velocity and *R* is the distance from the 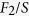 interface. The latter can convert 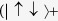

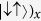
 into the parallel spin-triplet pairs 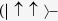

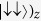
3. These parallel spin pairs will penetrate coherently over a long distance into the 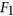 layer. So the transport of 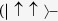

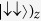
 can make a significant contribution to superharmonic Josephson current. Meanwhile, the period of this current becomes *π* and satisfies the second harmonic current-phase relation 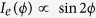
22,24. By contrast, in the Josephson junction with ferromagnetic trilayer only spin-triplet pairs 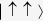 (or 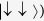 can transmit in central ferromagnetic layer, which provide the main contribution to the long-range first harmonic current^35^.

**Figure 4:**
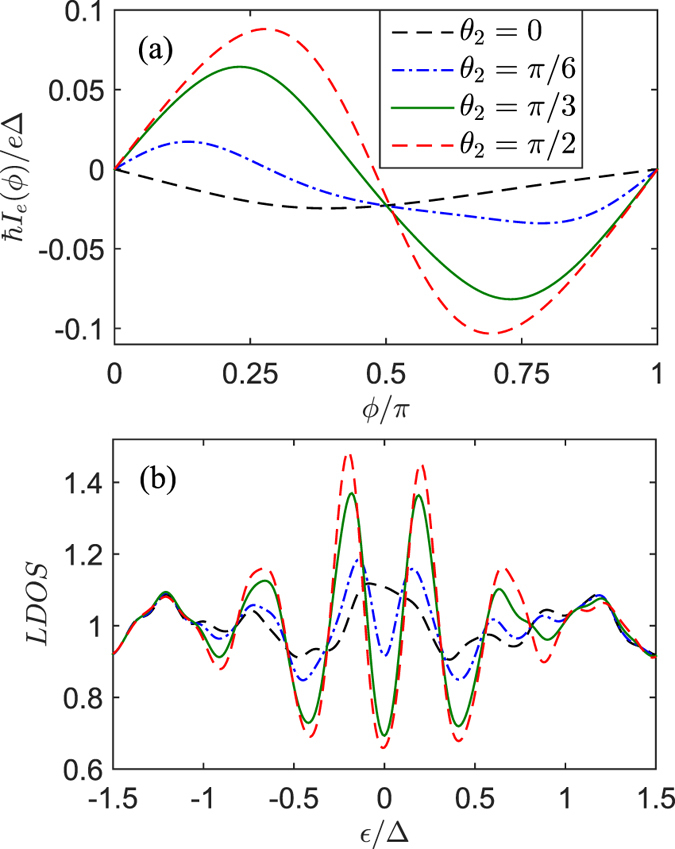
(**a**) the Josephson current-phase relation *I*_*e*_(*ϕ*) for four values of the relative angle *θ*_2_ between magnetizations. (**b**) The normalized LDOS in the *F*_1_ layer (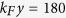) plotted versus the dimensionless energy 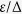 for different *θ*_2_, and the results are calculated at 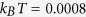. Other parameters are the same as in Fig. 3.

**Figure 5:**
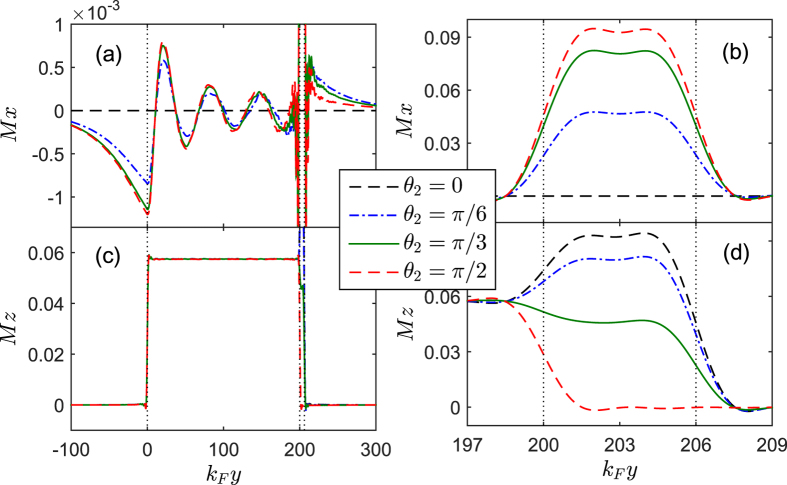
The *x* (top panels) and *z* components (bottom panels) of the local magnetic moment plotted as a function of the coordinate *k*_*F*_*y* for different *θ*_2_. The left panels show the behaviours over the extended *F*_1_ regions while the right ones show the detailed behaviours in the *F*_2_ layer. Other parameters are the same as in Fig. 3.

As plotted in Fig. 4, in the case of collinear orientation of magnetizations (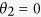), the current 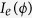 is weak enough and present a first harmonic feature. At this time, the long-range spin-triplet pairs 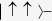

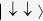
 are absent, so the LDOS in the 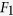 layer is almost equal to its normal metal value. With increasing 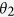, the magnitude of the second harmonic current is enhanced by the increased number of 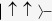

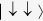
. Specifically, for orthogonal magnetizations (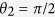), the second harmonic current grows big enough. Correspondingly, the LDOS is significantly enhanced with two distinguishable peaks. Moreover, the spatial profile of the local magnetic moments are plotted for several values of 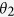 in Fig. 5. What’s most interesting is that the component 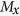 grows very quickly in the 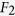 region with increasing 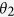, and also displays the penetration of the same component into the 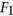 region. The induced 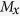 in the 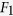 region does not only change magnitude as a function of position, but it also rotates direction. However, the component 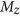 in the 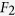 region will gradually decrease with 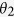 and remains almost unchanged in the 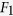 region.

**Figure 6:**
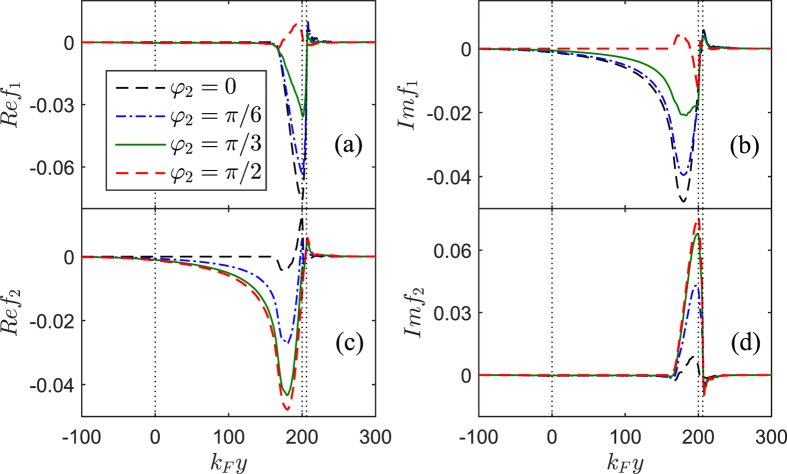
The spin-triplet pair amplitudes *f*_1_ [(**a**,**b**)] and *f*_2_ [(**c**,**d**)] plotted as a function of the coordinate 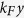 for several values of 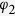 in the case of 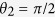. The left panels [(**a,c**)] show the real parts while the right ones [(**b,d**)] show the imaginary parts. Other parameters are the same as in Fig. 3.

**Figure 7:**
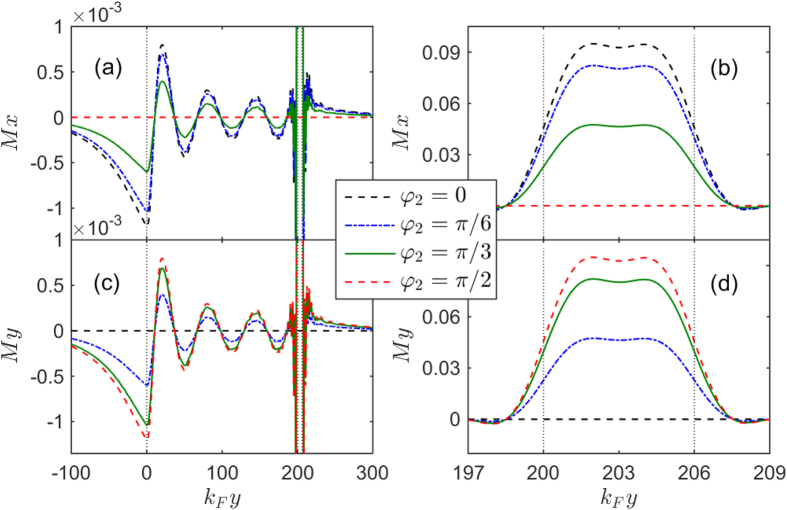
The *x* (top panels) and *y* components (bottom panels) of the local magnetic moment plotted as a function of the coordinate *k*_*F*_*y* for different *φ*_2_. The left panels show the behaviours over the extended *F*_1_ region while the right ones show the detailed behaviours in the *F*_2_ region. Other parameters are the same as in Fig. 3.

As stated above, the variation of the horizontal angle 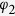 can not influence the Josephson current as the tilt angle 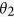 has a fixed value. However, the change of 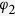 will induced a conversion of pairing fashion in the 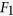 region. As shown in Fig. 6, on the condition of 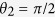, 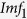 decrease gradually from a finite value to zero with increasing 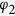, but 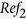 exhibit the opposite characteristics. These phenomena can be explained as follows: since the magnetic direction of the 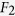 layer is oriented along the *x* axis (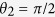, 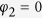), 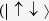 + 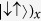 in the 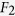 layer can be converted into 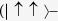

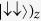
 in the 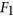 layer. In contrast, if the magnetic moment of the 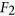 layer is along *y* axis (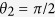, 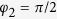), 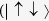 + 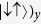 will be transformed into 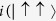 + 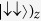, which can also penetrate into the 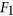 region a long distance and make a major contribution to the second harmonic current. At the same time, when the magnetization direction of the 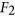 layer rotates from the *x* axis to the *y* axis, the induced magnetic moment in the 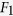 layer would correspondingly turn from 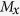 to 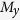, as seen in Fig. 7. In what follows, we focus on the dependence of the critical current on the thickness and exchange fields of two ferromagnetic layers under the condition of 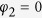.

### Superharmonic currents versus thickness and exchange field of the spin-active 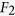 layer

**Figure 8:**
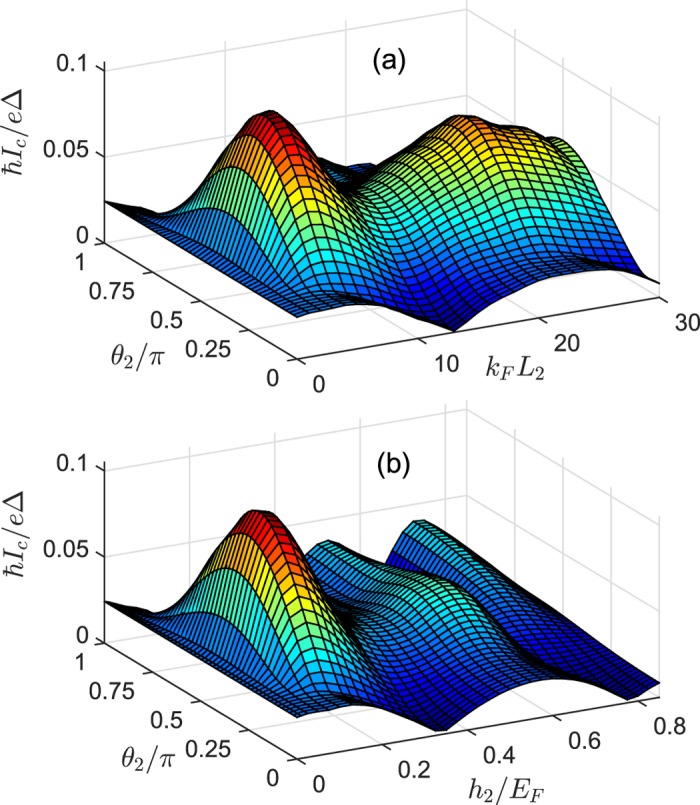
Critical current (**a**) as a function of 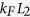 and *θ*_2_ for 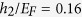, and (**b**) as a function of 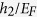 and *θ*_2_ for 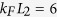. We set 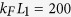, 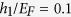, and 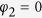.

Figure 8 shows the dependence of the critical current *I*_*c*_ on the length 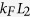 and exchange field 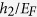 for different misalignment angle 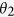 when the 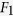 layer has fixed values 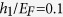 and 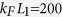. One can see that *I*_*c*_ is sufficiently weak and decays in an oscillatory manner in parallel (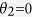) and antiparallel (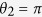) alignments of the magnetizations. This is because the exchange field in the *F*_2_ layer induces a splitting of the energy bands for spin up and spin down. This effect can make 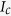 oscillate with a period 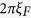 and simultaneously decay exponentially on the length scale of 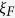
1. Here, 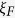 is the magnetic coherence length. In this case, only the spin-singlet pairs 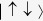 − 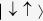 and spin-triplet pairs 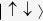 + 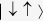 exist in the ferromagnetic layer. These two types of pairs can be suppressed by the exchange field of ferromagnetic layer and mainly provide the contribution to the first harmonic current.

On the other hand, if the orientations of the magnetic moments are perpendicular to each other (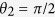), *I*_*c*_ also displays the oscillated behaviour with increasing 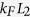, but its order of magnitude is larger than for collinear magnetizations. This characteristic behaviour can be attributed to the spatial oscillations of 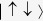 + 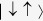 in the *F*_2_ region with period *Q* · *R*. It is well known that the Cooper pair in the *F*_2_ layer will acquire a total momentum *Q* because of the spin splitting of the energy bands. As described in ref. 36, for a fixed *Q* the amplitude of 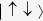 + 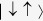 will vary with the length *R* (=*k*_*F*_*L*_2_) of the *F*_2_ layer. As a result, the oscillated 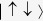 + 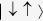 can be converted into 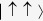 − 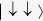 in the *F*_1_ layer by the spin-flip scattering, and then 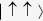 − 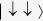 can propagate over long distance in the *F*_1_ layer and lead to the enhanced superharmonic current. Similarly, if one fixes 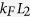 and changes 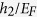, the same features about the critical current can be obtained (see Fig. 8(b)). It is worth mentioning that this oscillatory behaviour could be different from the oscillation of the critical current with the thickness of *F*_2_ layer in 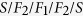 junction^36^, because the supercurrent in the central *F*_1_ layer derives from the contribution of 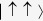 and manifests itself as a dominant first harmonic in the Josephson current-phase relation.

### Superharmonic currents versus length and exchange field of the long *F*_1_ layers

**Figure 9:**
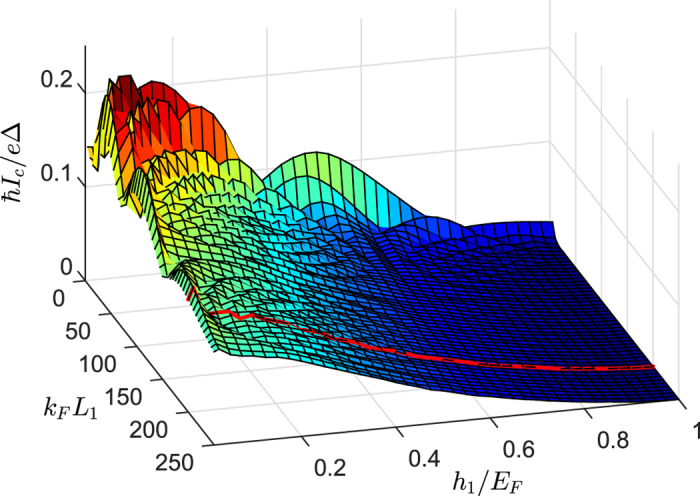
Critical current as a function of 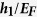 and 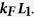 We set 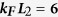, 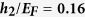, 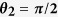, and 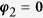.

In Fig. 9 the dependence of the critical current *I*_*c*_ on exchange field 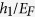 and length 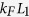 are plotted for 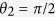. Compared with the Josephson junctions with homogeneous magnetization, *I*_*c*_ in this asymmetric junctions decreases slowly with increasing 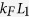 on the weak or moderate exchange fields. This feature illustrates that 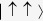 − 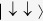 will propagate coherently over long distances in the *F*_1_ layer. Furthermore, *I*_c_ are almost monotonically decreasing with 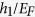 for various 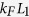 and will be prohibited completely at 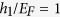. It indicates that the superharmonic current will be suppressed by the exchange field of the *F*_1_ layer. This phenomenon is clearly different from the first harmonic current in the half-metal Josephson junction with interface spin-flip scattering^9^,^16^, because the first harmonic current induced by 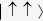 can not be suppressed by the exchange splitting.

**Figure 10:**
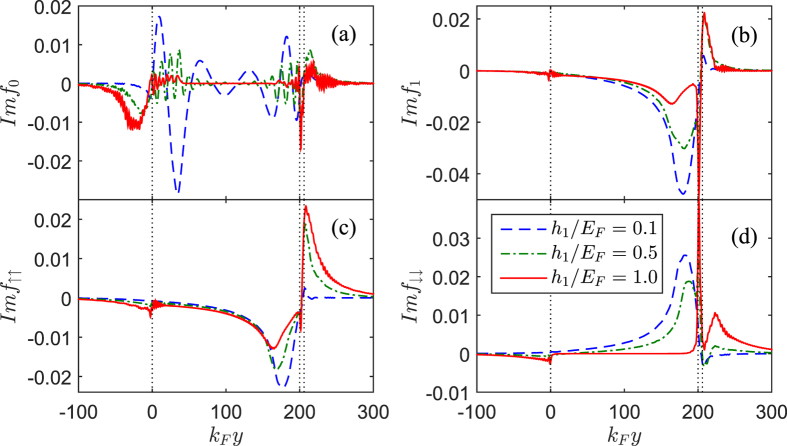
The imaginary parts of *f*_0_ (**a**), *f*_1_ (**b**), 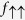 (**c**) and 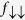 (**d**) plotted as a function of the coordinate 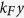 for several 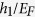. We set 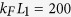, 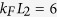, 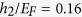, 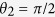, 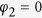, 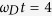, and 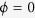.

**Figure 11:**
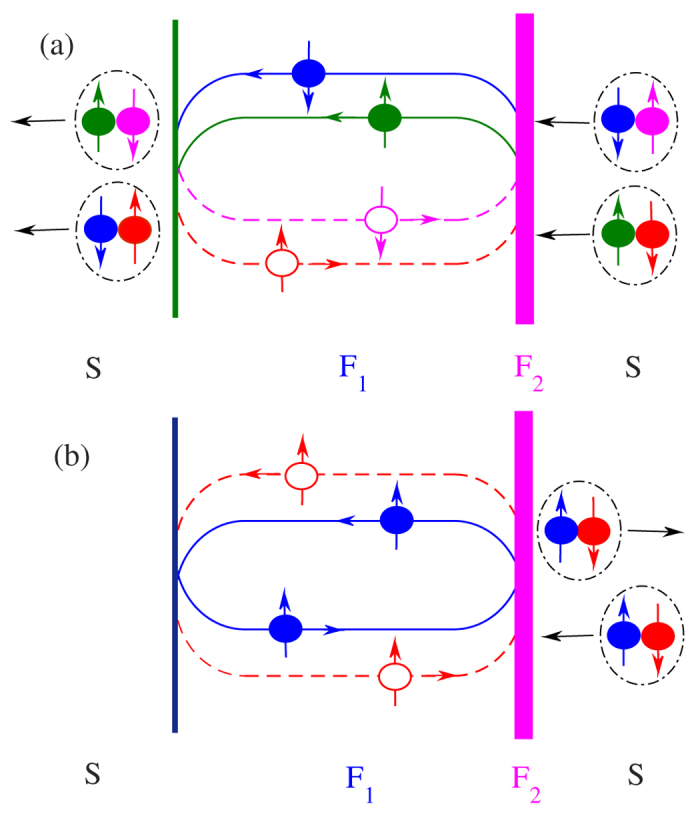
Two types of transference about the pairs of correlated electrons and holes. (**a**) The first one consists of two normal Andreev reflections occurred at 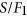 interface and two anomalous Andreev reflections at 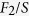 interface in the case of weak exchange field in the *F*_1_ layer. (**b**) The second one consists of two normal reflections at 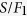 interface and two anomalous Andreev reflections at 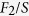 interface while the *F*_1_ layer is converted into half-metal.

In order to clearly explain the contribution of the spin-triplet pairs to the superharmonic current, we choose a fixed length 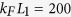 for discussion, as illustrated by the red line in Fig. 9. Under such conditions, we plot the distribution of the spin-triplet pairing functions *f*_0_, *f*_1_, 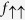 and 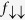 for three exchange fields 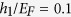, 0.5, and 1.0 in Fig. 10. With increasing 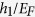, the magnitude of *f*_0_ and *f*_1_ in the *F*_1_ region are all reduced and *f*_0_ drops to zero at 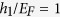. The reason can be summarized as follows: for weak exchange field 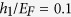 the triplet correlations 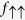 and 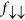 will generate in the *F*_2_ region and then combine into *f*_1_ in the F_1_ region. *f*_1_ decay spatially with approaching the 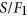 interface due to the fact that the pairs 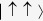 and 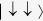 are recombined into the pairs 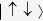 and 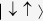 by the normal Andreev reflections. For 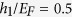, 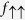 and 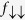 near the 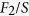 interface are both restrained. By contrast, 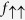 adjacent to the 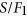 interface increases instead. Moreover, because 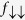 on the left side of *F*_1_ layer is suppressed, the recombination effect at the 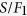 interface becomes weakened, in which case the superharmonic current will decrease. For a fully spin-polarized half-metal (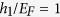), Fig. 10(d) shows that 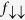 will be completely suppressed, but 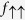 does not vanish and it’s magnitude seems to be a slight increase in the vicinity of the 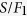 interface (see Fig. 10(c)). These characters can be attributed to the contributions from two important phenomena taking place at the 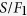 interface: normal Andreev reflections and normal reflections, as shown in Fig. 11 (a,b), respectively.

If the exchange field 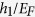 is weak enough, the normal Andreev reflections will mainly occur at the 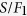 interface, which provide the main contribution to *I*_*c*_. In this case, the number of the pairs 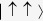 approximately equal to 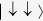, and then 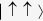 and 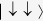 can combine into 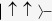

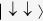
. Subsequently, 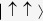 − 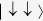 can be converted into 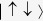 − 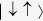 in the left *S*. With increasing 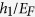, the normal Andreev reflections are gradually being replaced by the normal reflections, and the difference in the number of 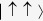 and 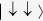 will enlarge simultaneously. As a result, the transition from 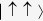 − 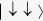 to 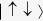 − 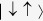 occurred at the 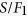 interface will be weakened. In the fully spin-polarized case (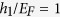) the absence of the spin down electrons makes it impossible to generate the normal Andreev reflections at 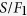 interface, and therefore the Josephson current is completely suppressed but 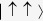 still exist. As depicted in Fig. 11(b), the electron transfer process is analogous to the unconventional equal-spin Andreev-reflection process reported in Ref. 37. Look at the whole picture, it is easy to understand the above process: 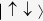 injecting from the right *S* is converted into 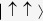 in the *F*_1_ layer, and 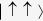 will be consequently reflected normally back as 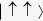 at the 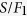 interface. Then 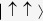 is transformed into 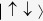 by the spin-flip scattering of the *F*_2_ layer. At last, 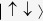 transports to the right *S*. In the whole process, none of Coopers can penetrate into the left *S*, so the Josephson current would be suppressed completely.

**Figure 12:**
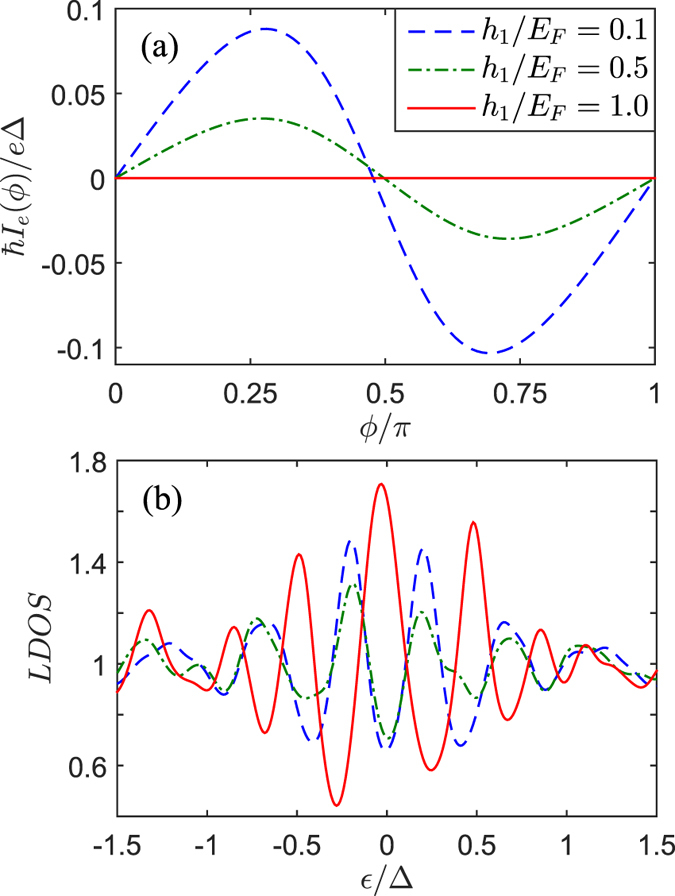
(**a**) the Josephson current-phase relation *I*_*e*_(*ϕ*) for different 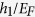. (**b**) The normalized LDOS in the *F*_1_ layer (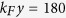) plotted versus the dimensionless energy 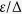, and the results are calculated at 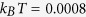. Other parameters are the same as in Fig. 10.

**Figure 13:**
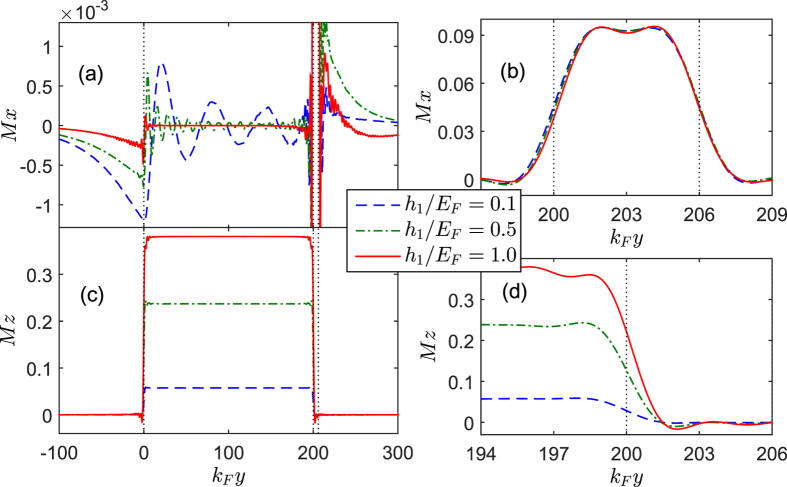
The *x* (top panels) and *z* components (bottom panels) of the local magnetic moment plotted as a function of the coordinate *k*_*F*_*y* for different *h*_1_/*E*_*F*_. The left panels show the behaviours over the extended *F*_1_ region while the right ones show the detailed behaviours in the *F*_2_ layer. Other parameters are the same as in Fig. 10.

In order to facilitate the experimental observations for the future, we plot the current-phase relation and the LDOS in the *F*_1_ layer at three points 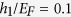, 0.5 and 1.0 in Fig. 12. With increasing 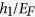, the superharmonic current 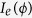 decreases and two distinguishable peaks in the LDOS will become weak correspondingly. It’s particularly noteworthy that if 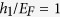 Josephson current was completely suppressed but the LDOS displays a sharp zero energy conductance peak which marks the presence of 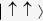. It can be measured in principle by STM experiments. And this feature is different from the conventional views: (i) The long-range triplet Josephson current is proportional to the parallel spin-triplet pairs 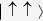 or 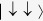. (ii) If the long-range triplet supercurrent passes through the Josephson junction, there will present the zero energy conductance peak in the LDOS of *F*. Finally, we discuss the influence of 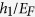 on the local magnetic moment. As can be seen from Fig. 13, in the *F*_1_ region *M*_*z*_ will grow with the increase of 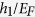, but the induced *M*_*x*_ could be suppressed. For 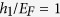, *M*_*z*_ reaches maximum but *M*_*x*_ will disappear. By contrast, *M*_*x*_ in the *F*_2_ region hardly changes with 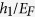, and *M*_*z*_ will partly permeate into the *F*_2_ layer.

To summarize, we have studied the long-range superharmonic Josephson current and the spin-triplet pairing correlations in the asymmetric 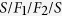 junction. We have shown that the superharmonic current was induced by the spin-triplet pairs 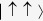 − 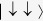 or 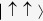 + 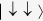 in the long *F*_1_ layer. The rotation of the magnetic moment in the thin spin-active *F*_2_ layer will not only modulate the amplitude of the superharmonic current through the junctions, but also realize the conversion from 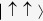 − 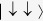 to 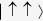 + 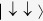 in the *F*_1_ layer. Besides, the critical current oscillates with the length and exchange field in the *F*_2_ layer. These features provide an efficient way to control the superharmonic current and the spin-triplet pairing fashion by changing the magnetic moment of the *F*_2_ layer. Specifically, the critical current almost decreases monotonically with the exchange field of the *F*_1_ layer, and if the *F*_1_ layer is converted into half-metal, the Josephson current disappear completely but the spin-triplet pairs 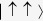 still exist within the entire *F*_1_ layer. This behavior is different from the conventional view about the relationship between the long-range current and the parallel spin-triplet pairs in the junctions with ferromagnetic trilayers. These results therefore indicated that the spin and charge degrees of the freedom can be separated in practice in the junction with ferromagnetic bilayers, and suggested the promising potential of these junctions for spintronics applications.

## Methods

The BCS mean-field effective Hamiltonian is given by^1^,^32^


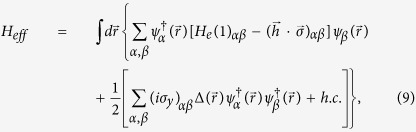


where 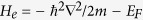 is the single-particle Hamiltonian, 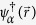 and 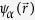 are creation and annihilation operators with spin *α*. 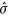 and *E*_*F*_ denote Pauli matrix and the Fermi energy, respectively. 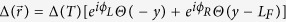 describes the superconducting pair potential with 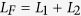. Here 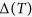 accounts for the temperature-dependent energy gap. It satisfies the BCS relation 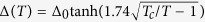, where 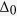 is the energy gap at zero temperature and *T*_*c*_ is the superconducting critical temperature. 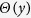 is the unit step function and 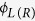 is the phase of the left (right) *S*.

By making use of the Bogoliubov transformation 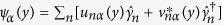 and the anticommutation relations of the quasiparticle annihilation and creation operators 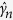 and 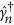, we have the Bogoliubov-de Gennes (BdG) equation^1^,^32^


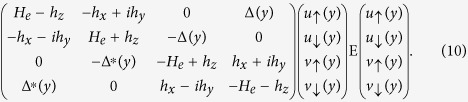


### Blonder-Tinkham-Klapwijk approach

The BdG equation (10) can be solved for each superconducting electrode and each *F* layer, respectively. For an incident spin up electron in the left *S*, the wave functions in the *S* leads and the *F*_*p*_ layer are


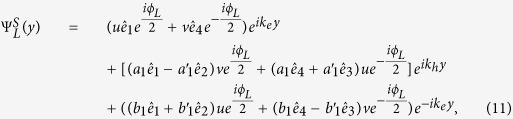



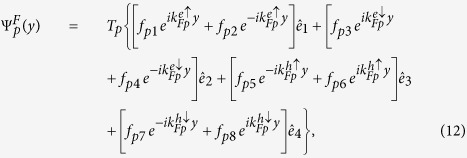



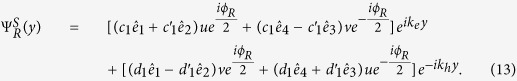


Here 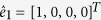, 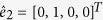, 
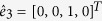
, 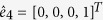 are basis wave functions. Quasiparticle amplitudes are defined as 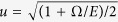 and 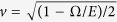 with 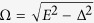. The perpendicular components of the ELQs (HLQs) wave vector in *S* leads and *F*_*p*_ layer are given by 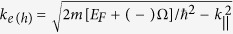 and 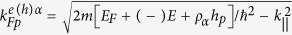 with 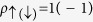, respectively. It is worthy to note that the parallel component 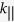 is conserved in transport processes of the quasiparticles. The matrix can be defined as^38^


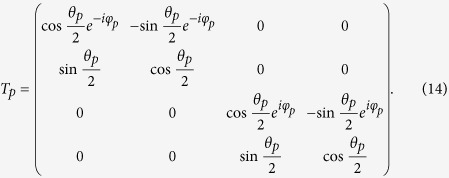


The coefficients 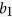, 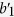, 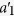 and *a*_1_ describe normal reflection, the normal reflection with spin-flip, anomalous Andreev reflection, and normal Andreev reflection, respectively. 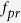 (*r* = 1–8) are quasiparticles wave function amplitudes in the *F*_*p*_ layer. Likewise, *c*_1_, *d*_1_, 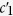 and 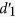 are the quasiparticles transmission amplitudes in the right superconducting electrode. All scattering coefficients can be determined by solving the continuity conditions of the wave function and its derivative at the interface


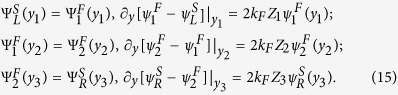


Here 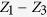 are dimensionless parameters describing the magnitude of the interfacial resistances. 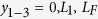 are local coordinate values at the interfaces, and 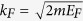 is the Fermi wave vector. From the boundary conditions, we obtain a system of linear equations that yield the scattering coefficients.

### Bogoliubov’s self-consistent field method

We put the 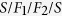 junction in a one-dimensional square potential well with infinitely high walls, then the eigenvalues and eigenvectors of the BdG equation (10) have the following changes: 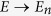 and 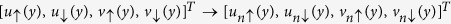. Accordingly, the corresponding quasiparticle amplitudes can be expanded in terms of a set of basis vectors of the stationary states^39^, 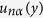 = 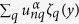 and 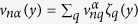 with 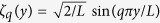. Here, *q* is a positive integer and 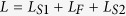. *L*_*S*1_ and *L*_*S*2_ are the thicknesses of the left and right superconducting electrodes, respectively. The superconducting pair potential in the BdG equation (10) is determined by the self-consistency condition^32^


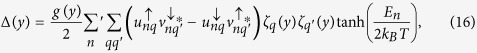


where the primed sum of *E*_*n*_ is over eigenstates corresponding to positive energies smaller than or equal to the Debye cutoff energy *ω*_*D*_, and the superconducting coupling parameter *g*(*y*) is a constant in the superconducting regions and zero elsewhere. Iterations are performed until self-consistency is reached, starting from the stepwise approximation for the pair potential.

## Additional Information

**How to cite this article**: Meng, H. *et al.* Long-range superharmonic Josephson current and spin-triplet pairing correlations in a junction with ferromagnetic bilayers. *Sci. Rep.* **6**, 21308; doi: 10.1038/srep21308 (2016).

## Figures and Tables

**Figure 1:**
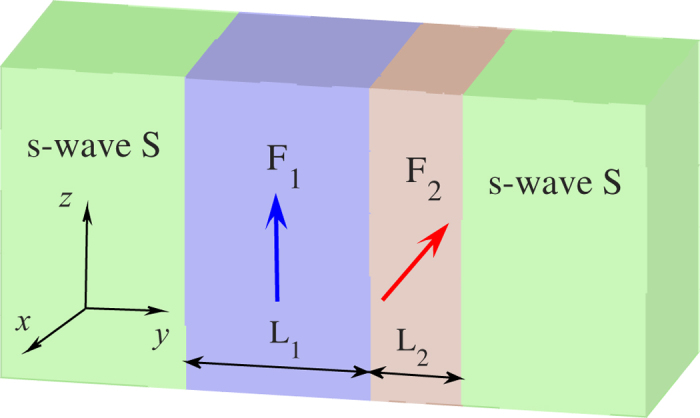
Schematic illustration of the *S*/*F*_1_/*F*_2_/*S* Josephson junction containing a bilayer ferromagnet. Thick arrows in *F*_1_ layer and *F*_2_ layer indicate the directions of the magnetic moments. The phase difference between the two s-wave *S*s is 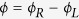.
